# Electromechanical impedance based self-diagnosis of piezoelectric smart structure using principal component analysis and LibSVM

**DOI:** 10.1038/s41598-021-90567-y

**Published:** 2021-05-31

**Authors:** Xie Jiang, Xin Zhang, Tao Tang, Yuxiang Zhang

**Affiliations:** 1 Xi’an Research Inst. of Hi-Tech, Xi’an, 710025 China; 2 Northwest Institute of Nuclear Technology, Xi’an, 710024 China

**Keywords:** Energy science and technology, Engineering

## Abstract

The long-term use of a piezoelectric smart structure make it difficult to judge whether the structure or piezoelectric lead zirconate titanate (PZT) is damaged when the signal changes. If the sensor fault occurs, the cases and degrees of the fault are unknown based on the electromechanical impedance method. Therefore, after the principal component analysis (PCA) of six characteristic indexes, a two-component solution that could explain 99.2% of the variance in the original indexes was obtained to judge whether the damage comes from the PZT. Then LibSVM was used to make an effective identification of four sensor faults (pseudo soldering, debonding, wear, and breakage) and their three damage degrees. The result shows that the identification accuracy of damaged PZT reached 97.5%. The absolute scores of PCA comprehensive evaluation for structural damages are less than 0.5 while for sensor faults are greater than 0.6. By comparing the scores of the samples under unknown conditions with the set threshold, whether the sensor faults occur is effectively judged; the intact and 12 possible damage states of PZT can be all classified correctly with the model trained by LibSVM. It is feasible to use LibSVM to classify the cases and degrees of sensor faults.

## Introduction

As one of the smart structures, the piezoelectric smart structure which uses the intelligent monitoring material^1^, piezoelectric ceramics as sensor and actuator has been widely used in engineering practice for its outstanding advantages such as high-cost performance, long-term stability, and online-monitoring capability^2, 3^. Some smart devices have been proposed to improve the monitoring performances of concrete structures, such as smart probes^4^, wearable devices^5^, or smart aggregate^6^. By embedding transducer in the host structure, the researcher can ensure high repeatability of the impedance signature as the transducer is always in a stable state. While to most structures like tank, pipeline, shell, etc. sensors can only be surface-mounted on monitored structures to measure impedance responses which may cause sensor faults during monitoring.

To date, a lot of researches on piezoelectric smart structure have been carried out based on the good piezoelectric lead zirconate titanate (PZT) patch properties^7–11^. With the popularization and service life extension of piezoelectric smart structure in practical structural health monitoring (SHM), the influence of the sensor state on measurement results has become a problem that cannot be neglected. Due to the long-term working of PZT in the open-air environment, the influence of external impact load, environmental erosion, improper operation of personnel, and other adverse factors may lead to breakage, debonding, pseudo soldering at the welding spot. Besides, the harsh natural environment, like a sandstorm, may cause the surface wear of PZT with continuous polish by gravel in wind. These defects will result in PZT degradation or even failure. After obtaining the impedance signal, if we do not diagnose prior and excluded the failing sensor but attribute the signal change to the structure damage, the real structure state information will be corrupted or even misjudged. Therefore, fault diagnosis of PZT is an important premise to ensure the effective operation of the SHM system.

Researches involved with PZT fault diagnosis and validation are classified into two main methods. The first method is mainly based on the ultrasonic guided wave (such as Lamb wave) as the effects of the defects are found to be significant, modifying the magnitude and the shape of the propagated waves. Liang et al.^12^ proposed a method of self-diagnosis and self-reconstruction of piezoelectric actuator and sensor network based on Lamb wave to realize real-time and reliable assessment of large-scale structure damage. Park et al.^13, 14^ studied the influence of bonding defects between PZT and structure on high-frequency SHM technology by using the Lamb wave propagation and impedance method, then verified the feasibility of the proposed sensor diagnosis program through experiments. Mulligan et al.^15^ evaluated and compensated the degradation index of the adhesive layer of PZT and used the lumped parameter model to monitor the degradation of the adhesive layer. They then used a pitch-catch configuration to discriminate against the effect of the bonding degradation on actuation and sensing. The second method relies on the characterization change of the electromechanical impedance (EMI) spectrum. Girgiutiu et al.^16^ designed a self-test program for sensors. When the sensor debonding occurs can be determined by extracting the characteristics of simultaneous occurrence and disappearance of sensor resonance and structural resonance. Huynh et al.^17^ proposed a new impedance model of a piezoelectric interface-driven system and proved the feasibility of this model for sensor diagnosis and structural integrity assessment. It was concluded that the root means square deviation (RMSD) of reactance and the slope of the admittance curve are able to distinguish structural damage from sensor debonding and fracture. Grisso and Inman^18^ built a prediction model for the slope of the susceptance and realized the accurate diagnosis of the sensor in a limited temperature range from 15 to 65 °C. Taylor et al.^19^ reviewed the diagnostic results of sensors in the dynamic loading test of wind turbine rotor blades by analyzing the slope of the electrical signal. Zheng et al.^20^ studied the effectiveness of the self-diagnosis technology of the piezoelectric sensor working in a harsh temperature environment and concluded that the self-diagnosis technology based on capacitance can detect sensor delamination and cracking at room temperature and liquid nitrogen temperature. Ai et al.^21^ used the real part of admittance to effectively identify structural damage and sensor debonding, breakage, and scratch. By using the resonance frequency shift method, they evaluated the detection ability of the damaged sensor and concluded that the damage to the sensor will have a serious impact on structural health monitoring. Overly et al.^22^ identified the degradation of mechanical/electrical properties and bonding defects of PZT by tracking the capacitance reflected by the imaginary part of admittance. After studying the consistency of long-term monitoring signals of PZT and the environmental effects, temperature effects, and bonding effects, Yang et al.^23^ concluded that interference signals in the high-frequency range (200 kHz–1 MHz) can distinguish PZT damages from structural damages. Park et al.^24^ applied the change of admittance to the identification of mechanical/electrical properties and debonding defects of piezoelectric wafers, which were verified by impact tests of composite plates.

There are many limitations for sensor fault diagnosis based on Lamb wave propagation in practical application. For example, it is difficult to select the appropriate center frequency for Lamb wave excitement which is determined by the specific environment; As the Lamb wave can only propagate in the plate and shell structure, the diagnosis effect may be poor when sensors are on the complex structure; The method based on Lamb wave often relies on sensor network which requires large numbers of PZT patches as actuators and sensors respectively. To build a sensor network, PZT patches should be mounted on the surface of the structure with a specific layout. As for the electromechanical impedance method, wave propagation can be excited at a wide range of frequencies. Since the signal can be self-generated and self-received with one transducer, fewer PZT patches are needed and the arrangement of the PZT array on the structure is more flexible. For the above reasons, we adopted the electromechanical impedance method for sensor fault diagnosis in the study.

In the electromechanical impedance method mentioned above, Ai, Overly, and Park et al. all found the different characteristic change laws of the impedance caused by various sensor faults. However, it is still difficult to identify what kind of sensor fault occurred when the characteristic change as there is not a one-to-one match between characteristic change and fault. Moreover, there may be more than one characteristic change in the actual signal compared with the benchmark. We focus on the extraction of useful information from the various characteristic changes to identify the damage cases and degrees from the impedance. In this paper, four cases of faults and their three fault degrees are introduced to the PZT patches on the aluminum plate. The structural damage and PZT fault are distinguished after the principal component analysis of the extracted impedance spectrum characteristics. Then we use the multi-classification model trained by LibSVM to identify the damage cases and degrees of PZT faults. The results of the study aim to make the identification of PZT more effective and further improve the accuracy of SHM.

## Detection technology based on electromechanical impedance method

**Figure 1:**
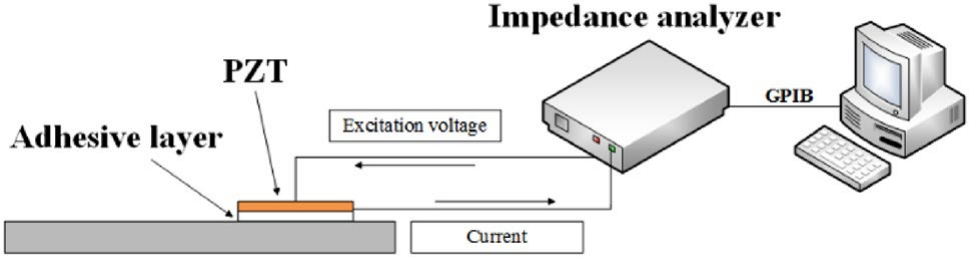
Schematic diagram of plate damage detection experimental devices.

The basic principle in the plate monitoring with the EMI method is^25, 26^: taking the positive and inverse piezoelectric effect of PZT as the sensor and the actuator respectively. A high-frequency AC voltage is generated by the impedance analyzer and applied to the PZT sensor which is coupled with structure through the adhesive layer. The deformation of PZT vibrates the tested structure which will, in turn, deform the coupled PZT and further cause the current generation. After the acquisition of the electrical impedance with the analyzer, the signal is transmitted to the computer by a general-purpose interface bus (GPIB) for signal characteristic extraction. The schematic diagram is shown in Fig. 1. We can infer the damage information of the tested structure and piezoelectric sensor with the comparison of the electrical impedance curves.

**Figure 2:**
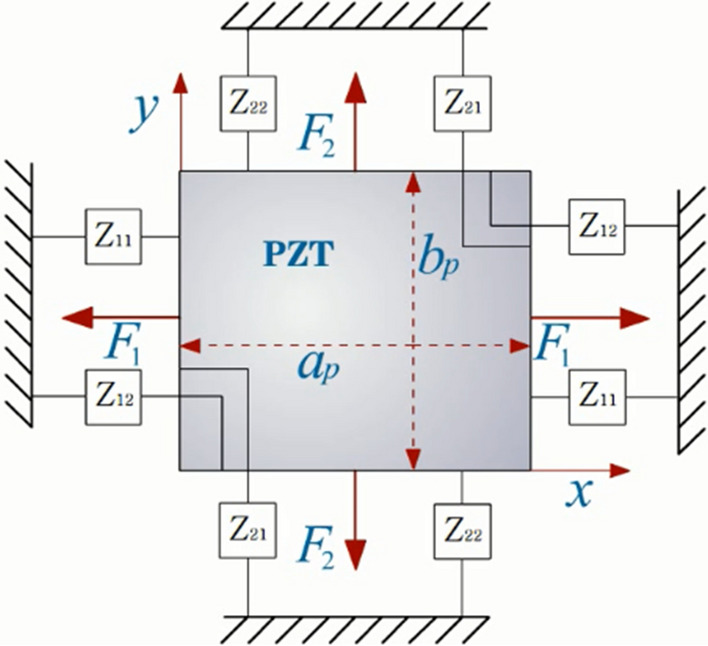
The two-dimensional EMI model.

As the inertial forces in two directions of the PZT are considered, it is more practical to analyze the plate which is a two-dimensional structure with the same dimension model. Two-dimensional EMI model and admittance expressions^27^ are shown in Fig. 2 and Eqs. (1)–(3).

1 $$Y(\omega ) = \frac{j\omega }{{h_{p} }}\left\{ {\frac{{\overline{Y}_{p}^{E} }}{{1 - \mu_{p}^{2} }}\left[ {\begin{array}{*{20}c} {\left( {d_{31} + \mu_{p} d_{32} } \right)\left( {2Ab_{p} \tan \frac{{K_{p} a_{p} }}{2} - d_{31} a_{p} b_{p} } \right)} \\ { + \left( {\mu_{p} d_{31} + d_{32} } \right)\left( {2Ca_{p} \tan \frac{{K_{p} b_{p} }}{2} - d_{32} a_{p} b_{p} } \right)} \\ \end{array} } \right] + \overline{\varepsilon }_{33}^{T} a_{p} b_{p} } \right\}$$

2 $$\left\{ {\begin{array}{*{20}l} A \hfill \\ C \hfill \\ \end{array} } \right\} = M^{ - 1} \left\{ {\begin{array}{*{20}l} {d_{31} } \hfill \\ {d_{32} } \hfill \\ \end{array} } \right\}$$

where

3 $$M = I - \frac{j\omega }{{\overline{Y}_{p}^{E} K_{p} h_{p} }}\left[ {\begin{array}{*{20}c} {\frac{{Z_{11} }}{{b_{p} }} - \frac{{Z_{21} \mu_{p} }}{{a_{p} }}} & {\frac{{Z_{12} }}{{b_{p} }} - \frac{{Z_{22} \mu_{p} }}{{a_{p} }}} \\ {\frac{{ - Z_{11} \mu_{p} }}{{b_{p} }} + \frac{{Z_{21} }}{{a_{p} }}} & {\frac{{ - Z_{12} \mu_{p} }}{{b_{p} }} + \frac{{Z_{22} }}{{a_{p} }}} \\ \end{array} } \right] \times \left[ {\begin{array}{*{20}c} { - \tan \frac{{k_{p} a_{p} }}{2}} & 0 \\ 0 & { - \tan \frac{{k_{p} b_{p} }}{2}} \\ \end{array} } \right]$$

In the Eqs. (1)–(3), Y*(ω)* = admittance; *j* = imaginary part of a complex number; *ω* = excitation angular frequency at work; $\mu_{p}$ = Poisson’s ratio of the PZT material; *b*_*p*_*, a*_*p*_, and *h*_*p*_ = length, width, and thickness of PZT; $\overline{\varepsilon }_{33}^{T}$ = $\varepsilon_{33}^{T} (1 - j\delta )$ = complex dielectric constant at constant stress *T*, $\varepsilon_{33}^{T}$ = dielectric constant, *δ* = dielectric loss factor; $\overline{Y}_{p}^{E}$ = $Y_{p}^{E} (1 + j\eta )$ = complex Young’s modulus of PZT at zero-elastic field, $Y_{p}^{E}$ = real Young’s modulus, *η* = mechanical loss factor; *d*_31_ and *d*_32_ = piezoelectric constants and *d*_31_ = *d*_32_ is usually assumed; *K*_*p*_ = $\omega \sqrt {\rho_{p} /\overline{Y}_{p}^{E} }$ = wave number, $\rho_{p}$ = mass density of the PZT; *A* and *C* = unknown coefficients determined from the boundary conditions. For a certain system, the above parameters are fixed values. *I* = identity matrix of 2 × 2; *Z*_11_ and *Z*_22_ = direct mechanical impedance of the structure; *Z*_12_ and *Z*_21_ = cross mechanical impedance of the structure. In a controllable environment of the laboratory, the states of PZT and adhesive layers are usually kept stable. The admittance is mainly affected by the mechanical impedance of the structure, including mass, stiffness, damping, and boundary conditions. Therefore, the change of admittance signal can be used as a diagnostic basis for structural damages^28–30^. However, the states of PZT and the adhesive layer will be possible to change in the actual environment. The admittance signal of the three-layer piezoelectric intelligent structure composed of piezoelectric wafers, an adhesive layer, and tested structure comprehensively reflects the state of both the structure and the PZT and the adhesive layer.

## Experimental investigation

### Experimental devices and procedure

**Figure 3:**
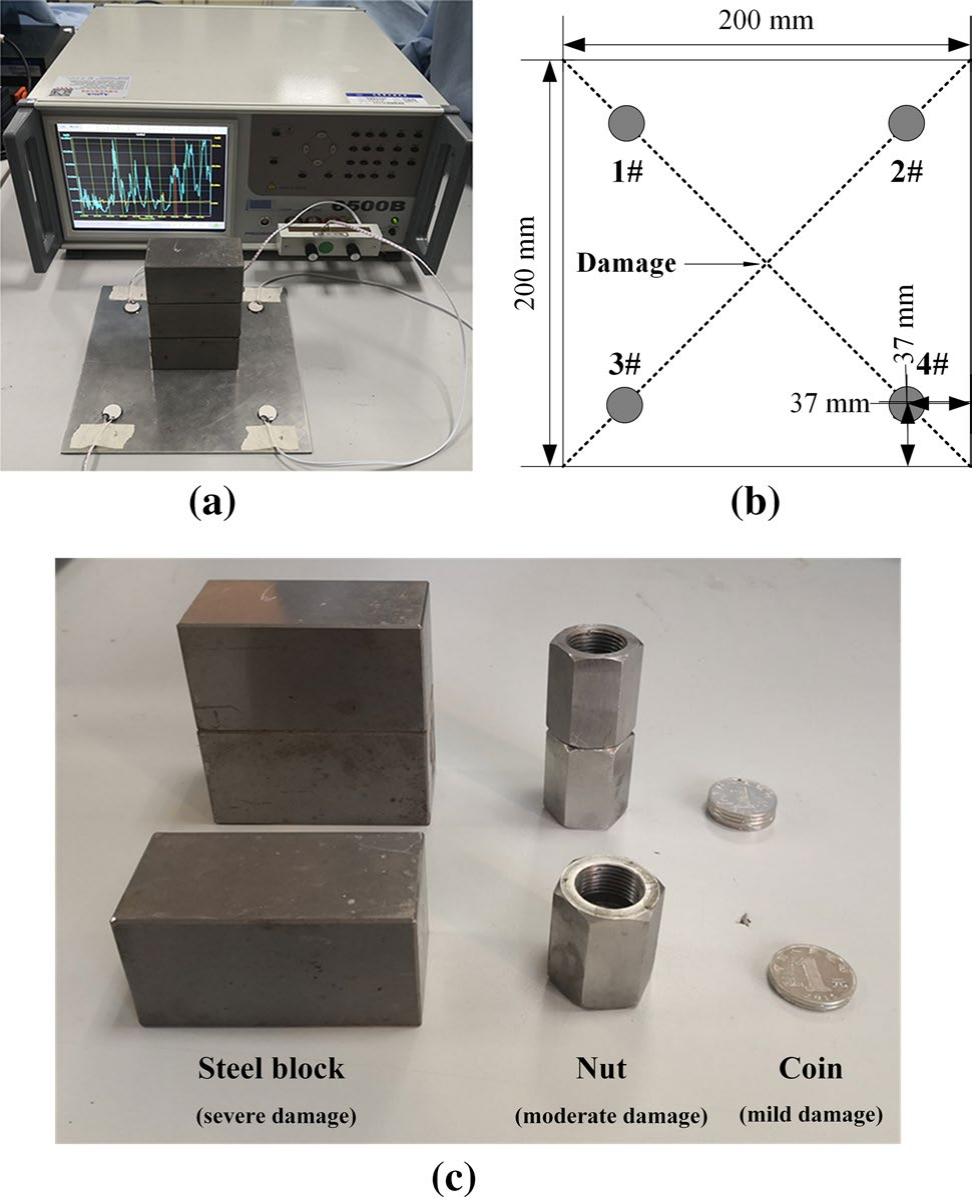
The experimental devices: (**a**) a WK6500B precision impedance analyzer and an aluminum plate with PZT whose layout diagram is shown in (**b**); (**c**) specimens used to simulate different cases of structural damages.

A designed experiment was conducted to explore the influence of structural damages and different sensor faults on the admittance spectrum. In the preparation step of the experiment, four PZT transducers (1–4#) were pasted on the diagonal symmetrical position of a square aluminum plate with modified acrylate adhesive. The adhesive has a mass ratio of epoxy resin and a hardener of 1:1. As shown in Fig. 3b, the distance between the center of each PZT and the center of the plate was the same. The 1#, 3#, and 4# PZT were bonded on the plate with the minimum feasible thickness of the adhesive layer to achieve the best detection effect^31^. At the same time, the thickness of the adhesive layer of 2# PZT was controlled at 0.5 mm to set the debonding condition conveniently. All of the PZT transducers were allowed to stand for 24 h until the epoxy adhesive solidified to make them closely fit.

As the mass addition will increase the local stress thus changing the mechanical impedance of the structure^21,32^, three different shapes and mass of specimens (i.e. the coin, nut, and cuboid steel block) were placed in the center of the plate to simulate different cases and degrees of structural damages, as shown in Fig. 3a,c. By controlling the same distance between the center of the weight and PZT transducers, the sensitivity of each PZT to structural damage is approximately the same^33^. In the study, four cases of sensor faults are introduced to PZT, including pseudo soldering, wear, breakage, and debonding. As pseudo soldering will lead to an increase of contact resistance between the wire and the PZT, to simplify the experiment, the levels of pseudo soldering are simulated by connecting different resistances in series with the positive electrode of the PZT. The material properties of PZT and plate are shown in Table 1.

**Table 1:** Material properties of PZT and plate.

Material property	Symbol	PZT	Aluminum plate	Unit
Model	–	PZT-5A	2A10	–
Mass density	$\rho$	7750	2750	kg/m^3^
Young’s modulus	*E*	65	70	Gpa
Poisson’s ratio	$\mu$	0.35	0.35	–
Piezoelectric constants	*d*_31_, *d*_32_	186	–	pC/N
Dielectric constant (*T* = 0)	$\varepsilon_{33}^{T}$	0.15	–	nF/N
Dielectric loss factor	$\delta$	0.02	–	–
Mechanical loss factor	$\eta$	0.001	–	–

When different specimens are stacked in the center of the plate, the amount of mass addition on the monitored structure and their corresponding working conditions are shown in Table 2. Three kinds of specimens when superimposed with different numbers were used to simulate different levels of mild damages, moderate damages, and severe damages. In the measurement, the positive and negative electrodes led out from the PZT surface were connected with the clamp of the WK6500B impedance analyzer. As a higher excitation voltage in a certain range significantly betters the detection sensitivity of the EMI method^33, 34^, we selected the upper limit excitation voltage 1 V from the range of 10 mV–1 V to make the detection more sensitive. For the choice of the frequency band ranges, a test on a wide frequency band of 10 kHz–1 MHz was carried out at first. In most experiments, 30–400 kHz is often used as the excitation frequency since it is sensitive enough to detect small changes in structural integrity^35, 36^. Considering the resonant frequency difference of each PZT, a comparatively wide frequency range of 30–130 kHz was selected as the resonant frequency after the trial and error method^37^. Since the high-frequency signal also contains damage identification information^23^, the non-resonant frequency band 130 kHz–1 MHz is studied as well. The specific experimental steps are as follows:

1. Obtain the admittance of four intact PZT transducers when the structure is in a healthy condition as the benchmark.
2. Measure the admittance of four intact PZT transducers under 1–9# structural damage condition respectively.
3. Set three levels of pseudo soldering, wear, debonding, and breakage in turn and collect the admittance signals when the structure is in a healthy state.

**Table 2:** Size and mass of experimental devices.

Specimen	Mass	Size	Stacked on the plate	Mass addition	Working condition
PZT	3 g	Φ16mm × 2 mm	–	–	–
Aluminum plate	232 g (with PZT)	200 mm × 200 mm × 2 mm	–	–	–
Coin	6 g	Φ25mm × 2 mm	2 coins	5%	1#
			4 coins	10%	2#
			6 coins	15%	3#
Nut	109 g	Internal diameter: M18mm × 1.5 mm External dimension: 28 mm Length: 35 mm	1 nut	47%	4#
			2 nuts	94%	5#
			3 nuts	141%	6#
Steel block	1012 g	40 mm × 40 mm × 80 mm	1 block	440%	7#
			2 blocks	880%	8#
			3 blocks	1320%	9#

**Figure 4:**
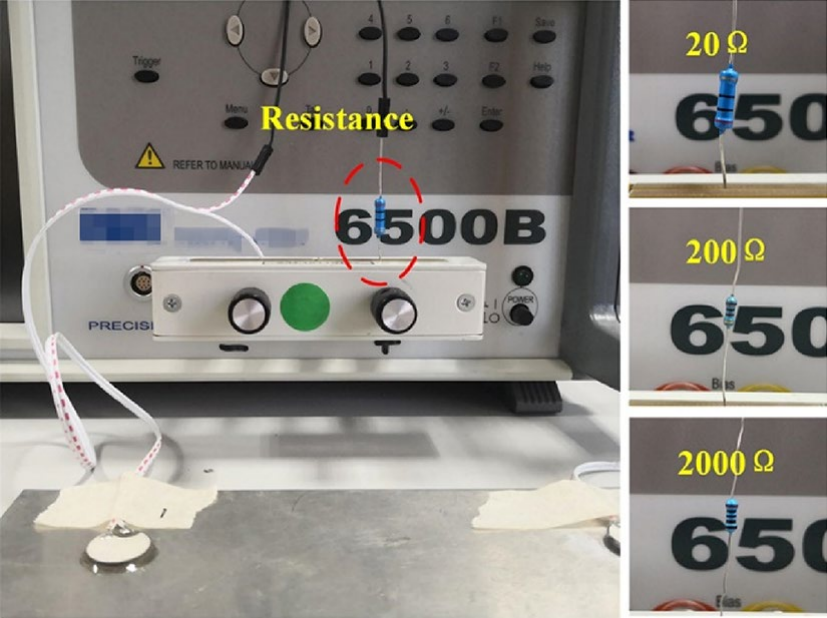
Pseudo soldering of 1# PZT including mild pseudo soldering (20Ω), moderate pseudo soldering (200Ω), and severe pseudo soldering (2000Ω).

**Figure 5:**
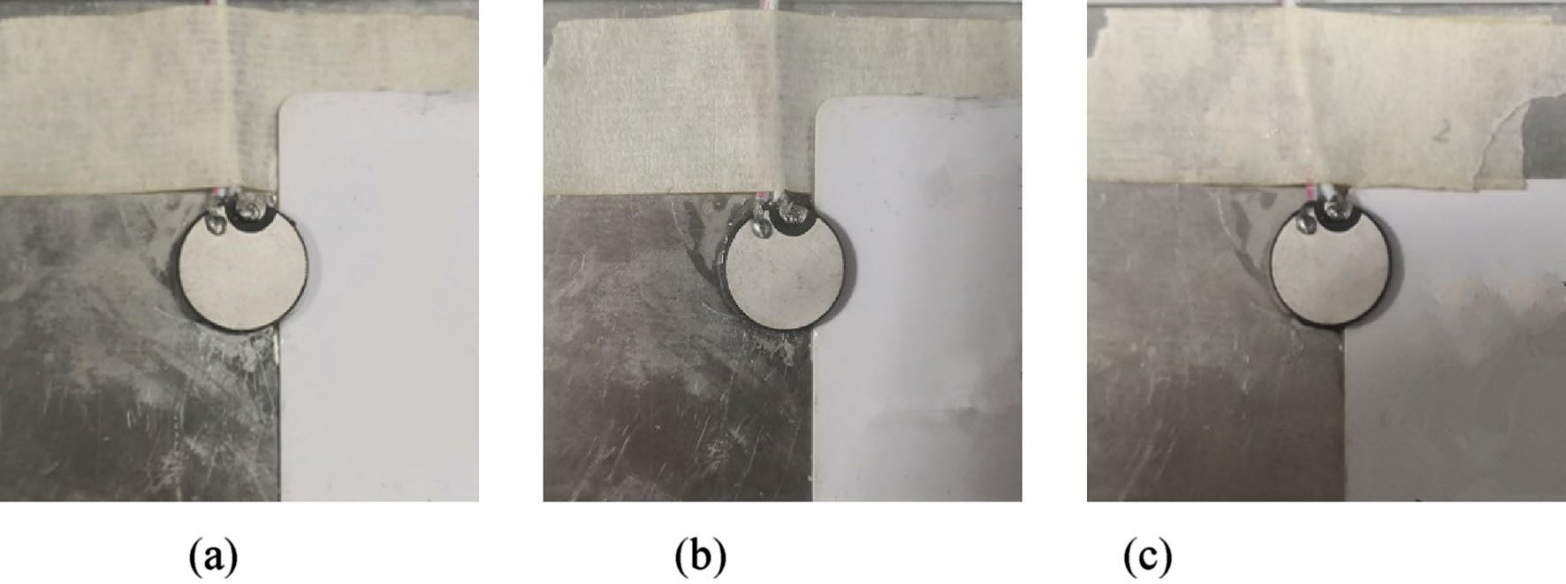
Debonding of 2# PZT: (**a**) 10% debonding; (**b**) 20% debonding; (**c**) 30% debonding.

**Figure 6:**
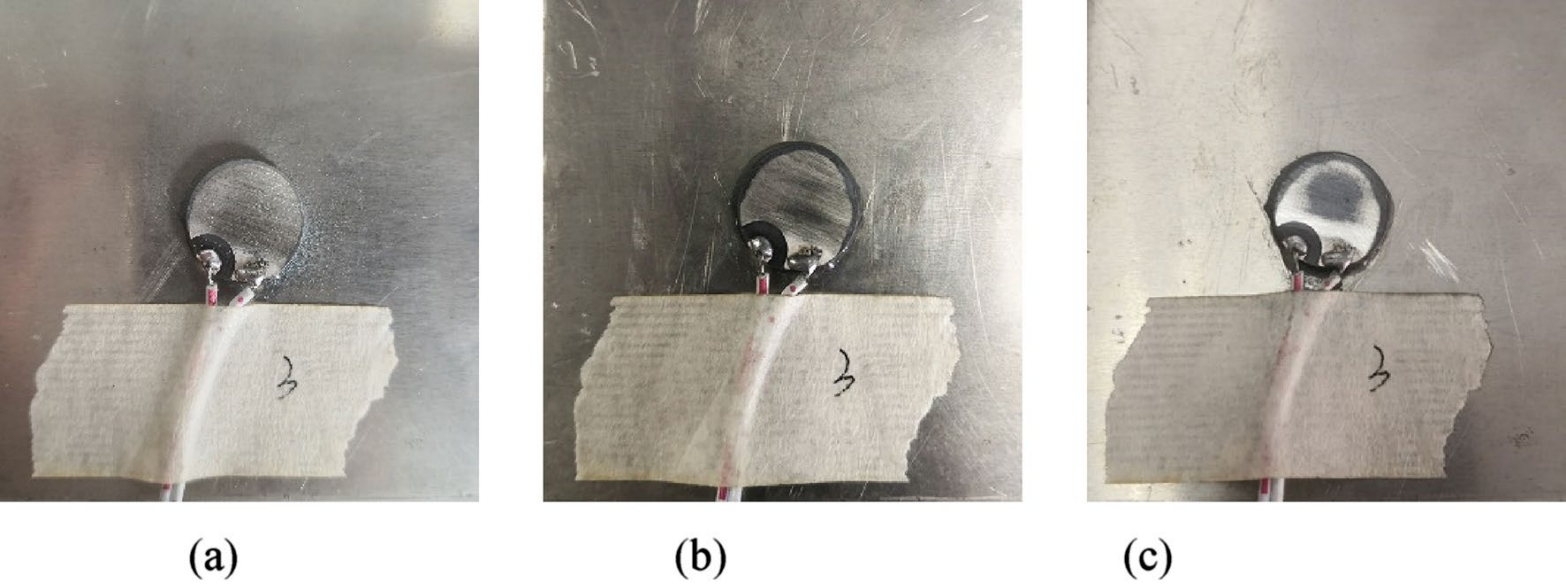
Wear of 3# PZT: (**a**) mild wear; (**b**) moderate wear; (**c**) severe wear.

**Figure 7:**
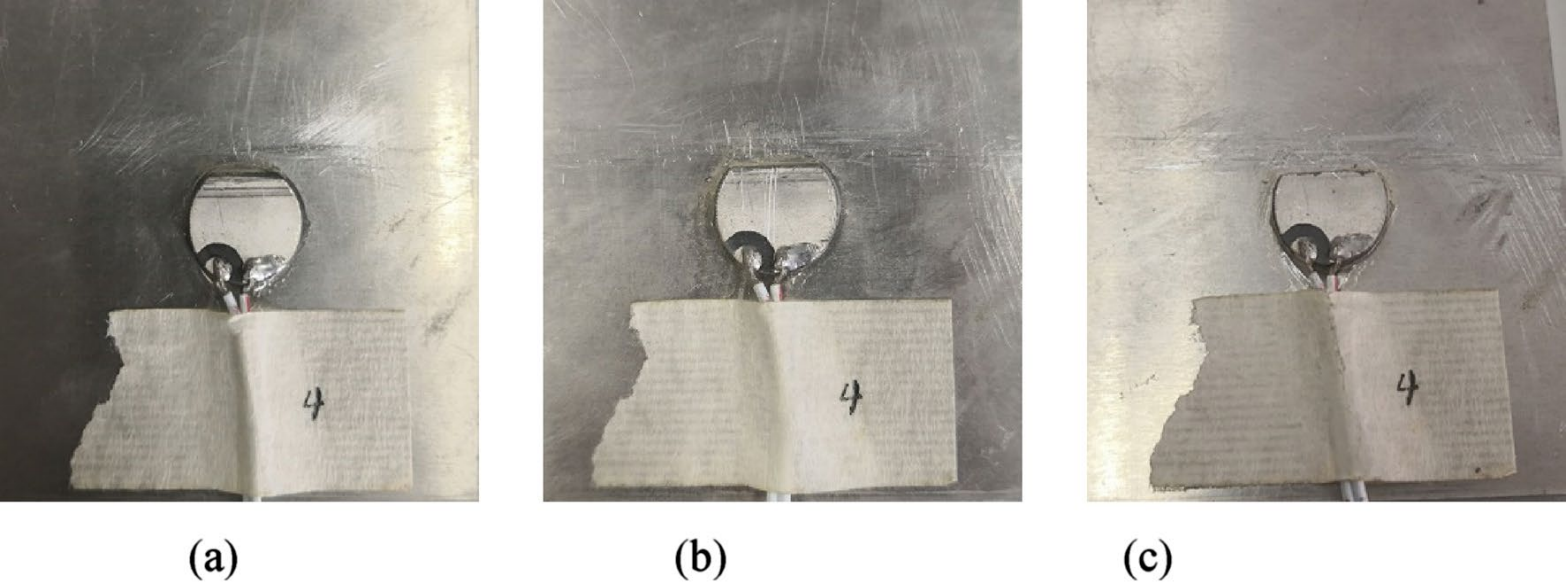
Breakage of 4# PZT: (**a**) 10% breakage; (**b**) 20% breakage; (**c**) 30% breakage.

Each group was measured five times and the average value was obtained to reduce the random error. Also, the experiment was conducted at a constant temperature of 26 °C to avoid the influence of temperature change on the signal. The experimental setup under different PZT faults is shown in Figs. 4, 5, 6, 7.

### Analysis of electromechanical admittance before and after PZT faults

**Figure 8:**
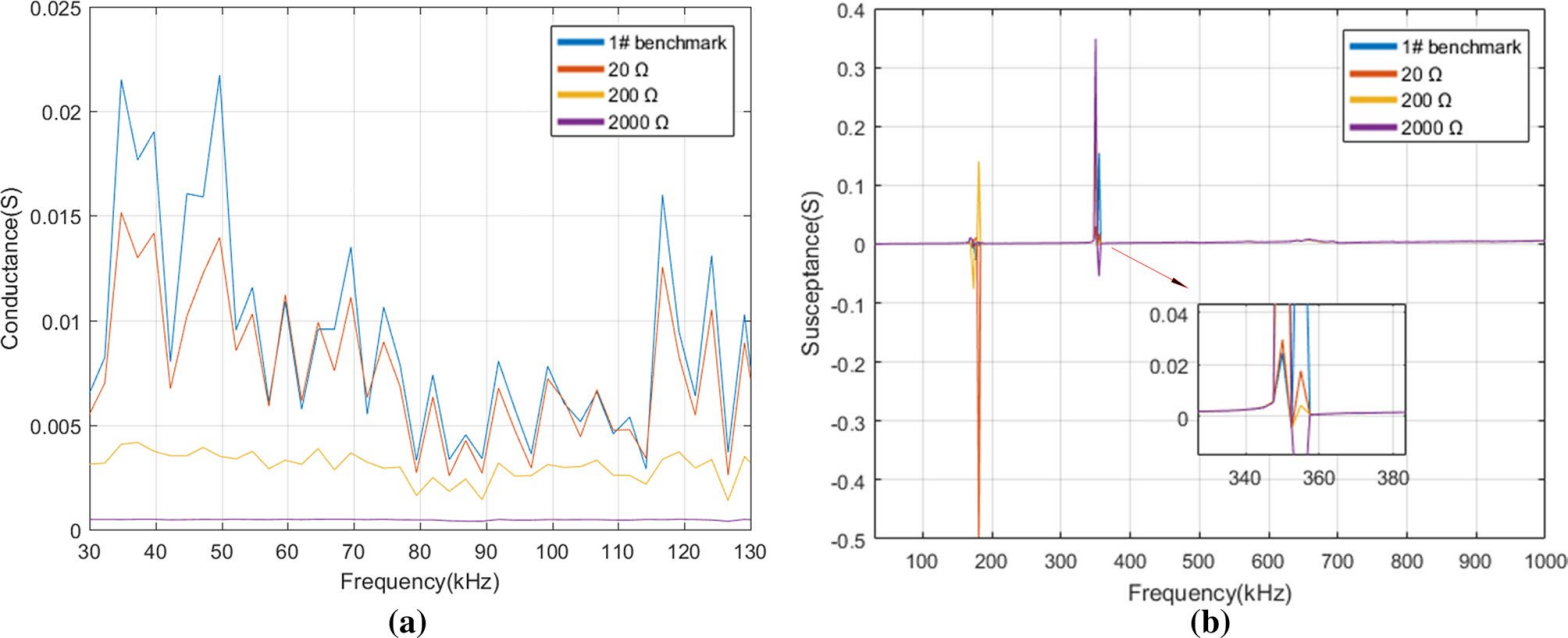
(**a**) The conductance and (**b**) the susceptance of 1# PZT under healthy and different pseudo soldering conditions.

After comparison, we found there are specific characteristic changes of conductance in the resonant frequency while not in the non-resonant frequency. Thus, the conductance at 30–130 kHz was displayed for illustrating the differences of conductance under various self-faults. As changes of susceptance are obvious in both resonant and non-resonant frequency, we focused on the susceptance in the frequency range of 30 kHz–1 MHz. Figure 8 depicts the real admittance (i.e. conductance) and the imaginary admittance (i.e. susceptance) of 1# PZT with three levels of pseudo soldering. It is observed in Fig. 8a that when 1# PZT is in the state of mild pseudo soldering, the magnitude of the resonant conductance peaks shows a reduction, meanwhile, the peak frequencies show no shift over the whole frequency band. The curve and benchmark are related closely to each other. As the degree of pseudo soldering keeps increasing, the conductance under different frequency bands drops gradually and the characteristics of conductance peak and trough are becoming less obvious. When in the state of severe pseudo soldering, the PZT cannot reflect the structural damages for its complete disappearance of the curve characteristics, which is regarded as the sensor failure. The susceptance in Fig. 8b shows two explicit resonant peaks under all pseudo soldering cases. From a partially enlarged view at 330–380 kHz, it is indicated that the defect of pseudo soldering will drop or rise the susceptance peak dramatically with the peak frequency unchanged.

**Figure 9:**
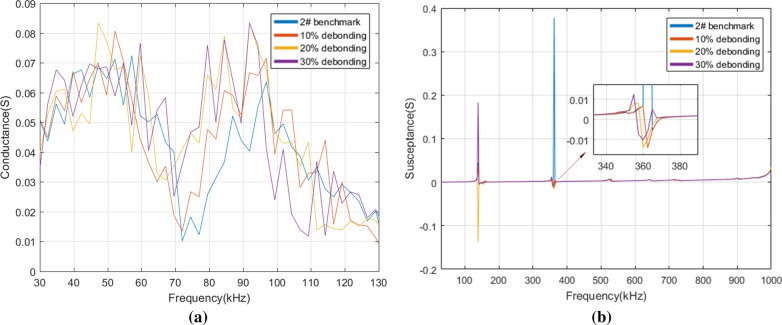
(**a**) The conductance and (**b**) the susceptance of 2# PZT under healthy and different debonding conditions.

For the PZT breakage cases, the conductance and the susceptance were measured in the frequency of 30–130 kHz, 30 kHz–1 MHz in Fig. 9. The observation from Fig. 9a shows that the conductance slightly increases as the debonding area rises from 0 to 30% in the frequency range of 75–82 kHz. Whereas, the characteristics are not so remarkable in other frequency bands. There are two susceptance peaks at 140 kHz and 360 kHz, see Fig. 9b. The growth of the debonding degree not only sharply reduces the magnitude of the peak at 360 kHz but also causes the leftward shift in the peak frequency.

**Figure 10:**
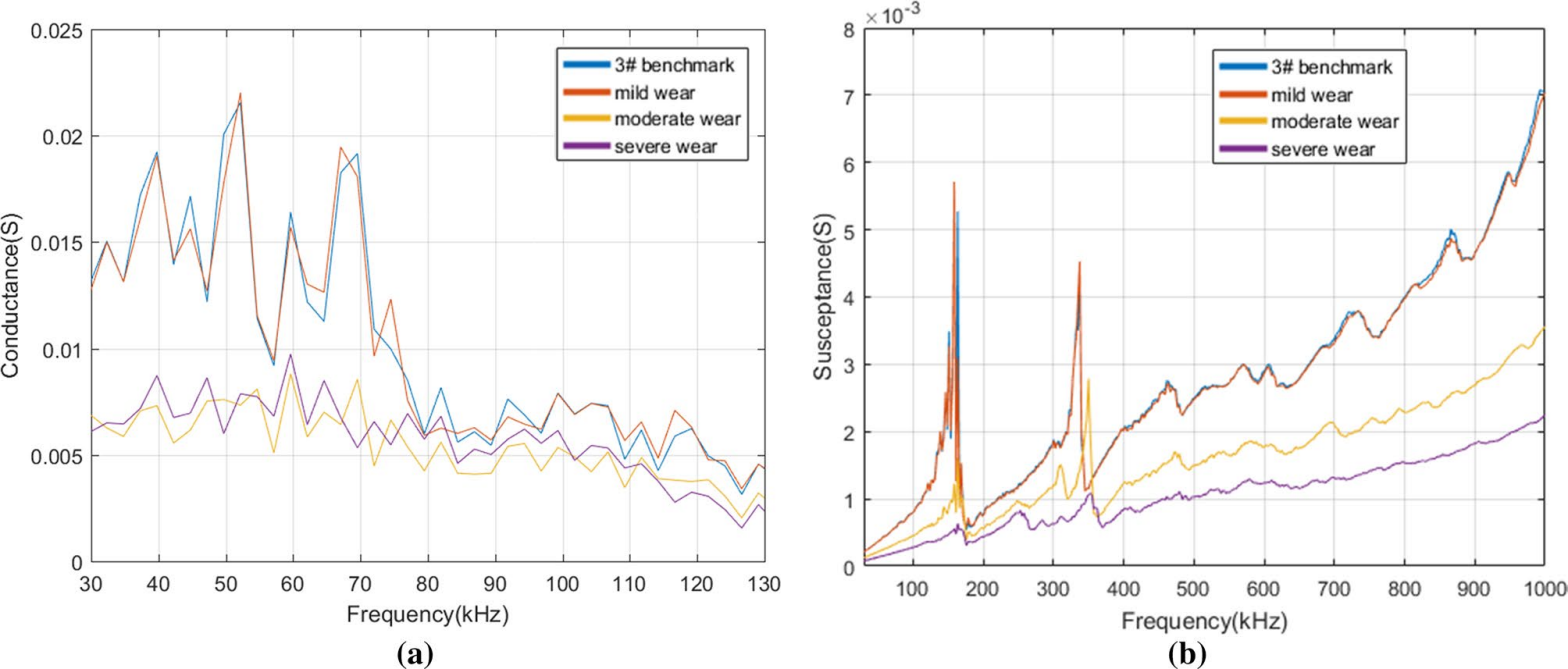
(**a**) The conductance and (**b**) the susceptance of 3# PZT under healthy and different wear conditions.

Figure 10 shows the conductance and the susceptance spectrum for different levels of wear. When the wear degree changes from mild to severe, the conductance decreases correspondingly. It is difficult to judge the occurrence of wear by a specific index as the change of the conductance curve does not show obvious regularity. As for the susceptance in Fig. 10b, the slope of the curve varies from the wear degree, which reduces gradually after the PZT surface is mildly, moderately, and severely worn. It also causes a slight rightward shift in resonant peaks at 158 kHz and 337 kHz.

**Figure 11:**
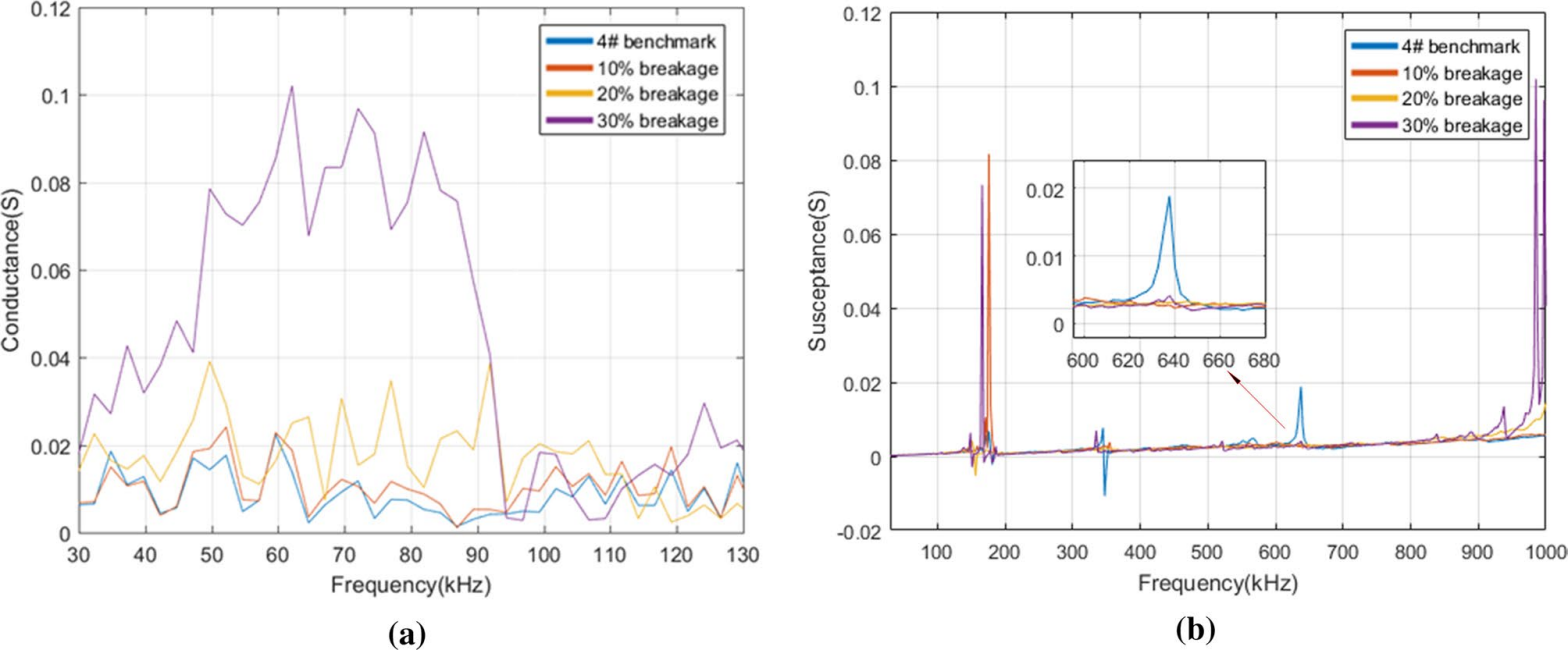
(**a**) The conductance and (**b**) the susceptance of 4# PZT under healthy and different breakage conditions.

According to Fig. 11a, the conductance increases gradually as the breakage area rises from 10 to 30% in the frequency range of 47–90 kHz. Figure 11b shows that some of the peaks disappear after breakage compared with the benchmark. The change of the conductance curve under the real experimental environment is inconsistent with the conclusion that breakage makes the conductance decrease drawn by Huynh^17^ which is based on the one-dimensional modified model. The reason is that the breakage not only changes the size of the effective bonding area but also produces the secondary vibration effect due to the different roughness of the damaged PZT edge. The experimental result shows that some extra peaks cannot be predicted by the model analysis^38, 39^ and it is difficult to extract the general rule from the electromechanical impedance curve change under damaged conditions.

From the above analysis, a conclusion can be reached that different PZT faults have different indication frequency bands and indication characteristics. It is known that the structural damage mainly causes the impedance change in the resonant frequency band while the sensor fault causes the impedance change in the resonant and non-resonant frequency bands^17^. In many studies, the main characteristic indexes of the impedance spectrum curve used to distinguish structural damages and PZT faults are as follows: the change of resonant conductance peak magnitude^40^, the peak frequency shift of conductance^40^, the slope of the susceptance linear fitting curve^13, 17, 40^, the change of resonant resistance peak magnitude^13, 17^, and RMSD of reactance in non-resonant frequency band^17^. RMSD^41, 42^, the statistical indicator, is often used to characterize the difference between two curves quantitatively whose expression is as follows:

4 $$RMSD = \sqrt {{{\sum\limits_{{N_{r} }} {\left[ {Im(Z_{i} ) - Im(Z_{i}^{0} )} \right]^{2} } } \mathord{\left/ {\vphantom {{\sum\limits_{{N_{r} }} {\left[ {Im(Z_{i} ) - Im(Z_{i}^{0} )} \right]^{2} } } {\sum\limits_{{N_{r} }} {\left[ {Im(Z_{i}^{0} )} \right]^{2} } }}} \right. \kern-\nulldelimiterspace} {\sum\limits_{{N_{r} }} {\left[ {Im(Z_{i}^{0} )} \right]^{2} } }}}$$

In this formula, $Im(Z_{i}^{0} )$ and $Im(Z_{i} )$ = imaginary part of the impedance before and after damage at the *i*th frequency point; *N*_*r*_ = number of measurement frequency points. Due to the performance difference between the PZT transducers, we tracked the slop change rather than the slope of the curve to ignore the adverse effects. To extract the information of curve spectrum more comprehensively in both resonant and non-resonant frequency bands, four characteristic indexes were selected, including the change of resonant conductance peak magnitude, the slope change of the susceptance linear fitting curve, the average shift of conductance peak frequency, and the RMSD of reactance in the non-resonant frequency band. The average shift of peak frequency refers to the average shift of the five bands (30–200 kHz, 200–400 kHz, 400–600 kHz, 600–800 kHz, 800 kHz–1 MHz) divided from the test frequency range to avoid inaccurate result caused by the improper selection of frequency range. Another two indexes, the area change rate of conductance in resonance and non-resonance frequency band were also introduced to evaluate the overall deviation of the curve. The six indexes coded as characteristic indexes 1–6#, in turn, were investigated in the study.

**Figure 12:**
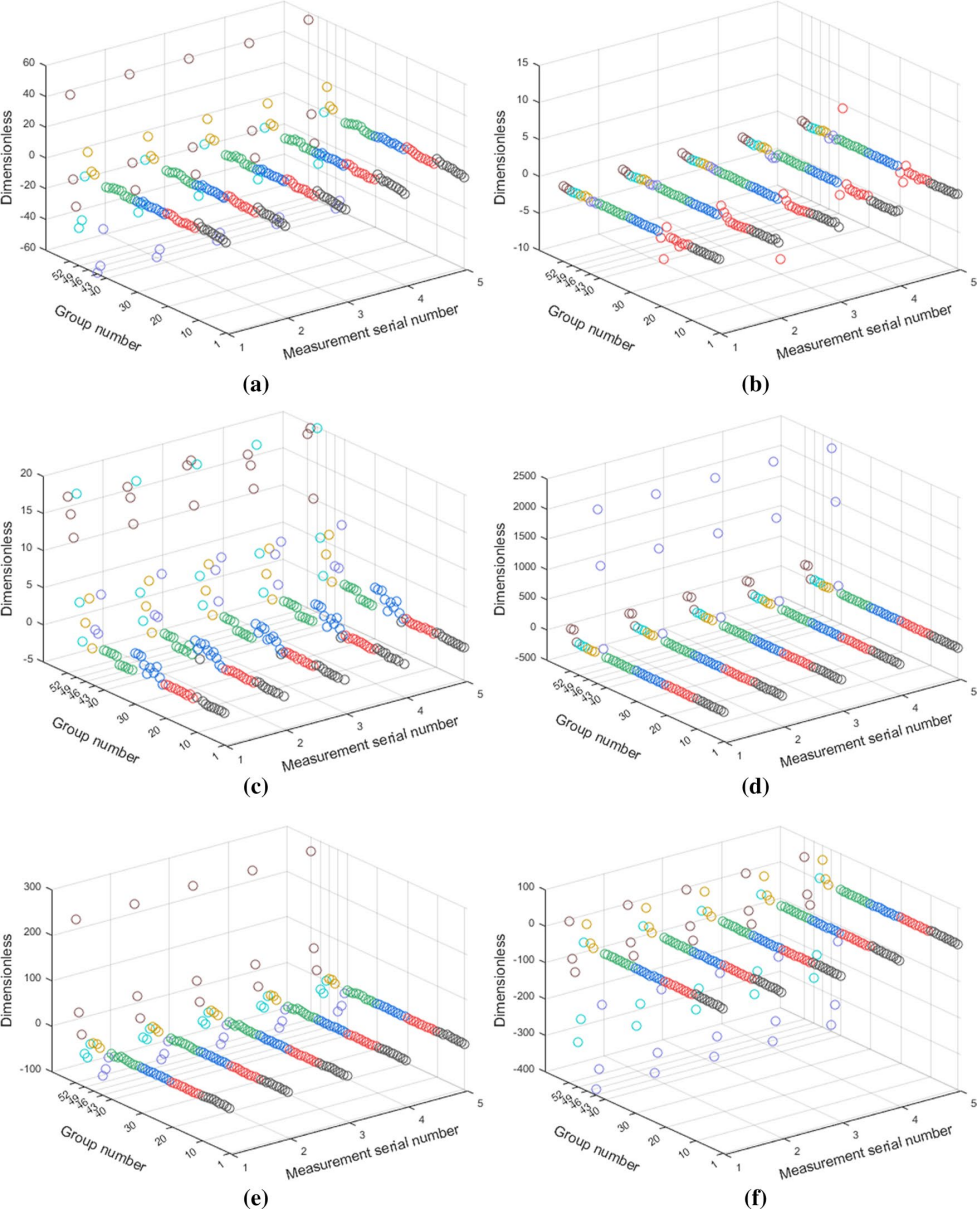
The indication effects of 6 characteristic indexes after standardization for different structural damages and PZT faults: (**a**) the change of resonant conductance peak magnitude (1# index); (**b**) the slope change of the susceptance linear fitting curve (2# index); (**c**) the average shift of conductance peak frequency (3# index); (**d**) the RMSD of reactance in the non-resonant frequency band (4# index); (**e**) the area change rate of conductance in the resonance frequency band (5# index); (**f**) the area change rate of conductance in the non-resonance frequency band (6# index).

The 1–6# indexes were used to evaluate the curves under various working conditions and the indication effects after standardization are shown in Fig. 12a–f. X coordinate represents the serial number of repeated groups and Y represents different experimental conditions. Among the 52 groups, 1–40 describes the working conditions when 1–4# PZT transducers are intact while 41–52 illustrates the working conditions that PZT transducers are damaged. In the first 40 groups, 1–10 represents the test data of no structural damage, three levels of mild structural damage, three levels of moderate structural damage, and three levels of severe structural damage. Similarly, groups 11–20, 21–30, and 31–40 are the data of 2–4# PZT transducers under corresponding structural damage conditions. The indication of groups 41–52 shows the data of 1–4# PZT transducers in different degrees of pseudo soldering, debonding, wear, and breakage conditions when the structure in a healthy state.

As a characteristic index for sensor self-diagnosis, it is supposed to meet the following two requirements: the index is effective in differentiating PZT faults from structural damages; the index has strong applicability and stability for different cases and degrees of damages. As indicated in Fig. 12a,e,f, 1#, 5#, and 6# indexes all fluctuate slightly around a different constant in groups 1–40 and change markedly in 41–52. Depend on the above requirements, 1#, 5#, and 6# indexes are suitable for the diagnosis of PZT state because of their stable identification effect when only structural damages occurred and the notable differentiation after the PZT faults. With the aggravation of damage degree of PZT, the indication effects become more significant. For Fig. 12b, the 2# index under different working conditions shows similar laws with the benchmark. The effect of the 2# index to distinguish the structural damages and the PZT faults with the admittance is not obvious. Figure 12c shows that four cases of PZT faults have changed the 3# index vastly. At the same time, there is also a large degree of deviation when moderate structural damage or severe structural damage occurs. In other words, only taking the 3# index as the distinguishing index will easily lead to the misjudgment of PZT and structure defects. In Fig. 12d, 4# indexes of groups 1–40 and 44–49 are similar while large offsets are shown in groups 41–43 and 50–52, thus leading to the conclusion that the 4# index is more sensitive to the wear and breakage of PZT transducers. The characteristic indexes of each PZT under different self-fault states are shown in Table 3.

**Table 3:** Characteristic indexes of PZT under different damage conditions.

Index	1# PZT			2# PZT			3# PZT			4# PZT		
	Mild pseudo soldering	Moderate pseudo soldering	Severe pseudo soldering	10% debonding	20% debonding	30% debonding	Mild wear	Moderate wear	Severe wear	10% breakage	20% breakage	30% breakage
1	− 28.68	− 53.51	− 59.38	4.06	4.73	16.65	− 0.30	− 29.75	− 35.79	− 22.64	− 5.74	51.17
2	− 0.11	− 0.08	− 0.19	0.12	0.22	0.06	0.05	− 0.03	− 0.07	0.02	0.13	0.25
3	8.14	2.23	3.93	− 0.11	6.47	3.00	0.42	4.90	19.56	12.81	17.72	18.32
4	113.60	1484.40	2372.31	4.07	1.10	3.49	16.84	12.52	14.54	26.38	210.99	203.32
5	− 10.28	− 40.06	− 57.17	1.79	6.11	5.87	− 0.49	− 29.68	− 25.96	12.44	57.72	262.01
6	− 144.88	− 323.66	− 382.46	− 0.24	10.40	59.63	4.79	− 210.50	− 276.38	− 86.46	− 54.32	44.58

Table 3 shows that with a gradual increase in the degree of pseudo soldering, 1#, 4#, 5#, and 6# indexes have the same trend of rise or drop and the average change rates of damage degrees are 49%, 633%, 166%, and 70%, among which the 4# index is the most sensitive index to the pseudo soldering of PZT. For sensor debonding, 1# and 6# indexes rise to 16.65 and 59.63 when the debonding level increases up to 30%. The 6# index has a better reflection of the debonding area as its average change rate of 1981% is greater than 134% of the 1# index. Likewise, it is suitable to choose the 6# index and the 1# index as the indicators of the PZT debonding and PZT wear, respectively. In conclusion, 1# and 6# indexes reflect the degrees of various PZT faults and the characteristic indexes’ sensitivity to different PZT faults are different.

## Judgment of PZT state based on principal component analysis

To explore the relationship between different characteristic indexes, the correlation coefficients are shown in Table 4.

**Table 4:** Correlation coefficients among the six indexes.

Index	1	2	3	4	5	6
1	1.000					
2	0.053	1.000				
3	− 0.130	0.025	1.000			
4	− 0.660	− 0.031	0.139	1.000		
5	0.679	0.052	0.475	− 0.191	1.000	
6	0.908	0.041	− 0.389	− 0.774	0.365	1.000

It can be seen from Table 4 that there is a certain correlation between each index, among which the 1# index has a significant positive correlation with the 6# index and moderate correlation with the 4# index and the 5# index. The correlation between the 2# index and other indexes is low. Additionally, there is a significant negative correlation between the 4# index and the 6# index. The information provided by each characteristic index overlaps and the description ability of each index to different damage is not the same. Therefore, it is not accurate to use all these indexes to identify structural damages and PZT faults directly. The sensitivity of six characteristic indexes to four damage cases is different and the frequency band which accurately reflects the characteristics of sensor faults cannot be determined in advance. By manually selecting the frequency band and analyzing the characteristics, it will not only cost a lot of manpower but also lead to a high probability of misjudgment when only a single index is used as the distinguishing standard. Given the above shortcomings, the principal component analysis method was used to extract the principal components with which it is easier to differentiate structural damages from PZT faults.

As a multivariate statistical method, principal component analysis (PCA) is able to separate and extract several orthogonal comprehensive indicators which completely reflect the different characteristics from many variable factors by reducing the dimension^43–45^. Generally, the extracted comprehensive index is called the principal component (PC), in which each principal component is a linear combination of original variables and is not related to each other. It can be expressed by Eqs. (5) and (6).

5 $${\mathbf{Y}} = {\mathbf{TX}}$$

6 $${\mathbf{XX}}^{T} = {\mathbf{A\Sigma }}^{2} {\mathbf{A}}^{T}$$

where Eq. (5) represents the process of converting the initial sample set ${\mathbf{X}} \in \Re^{m \times N}$ to a new coordinate system, i.e. the principal component diversity ${\mathbf{Y}} \in \Re^{n \times N}$. ${\mathbf{\rm T}} \in \Re^{n \times m}$ = transformation matrix = the *n* most important eigenvectors of the covariance matrix of ${\mathbf{X}}$; *N* = the total number of samples; *m* and *n* = the number of variables in the initial sample set and the principal component diversity set; Eq. (6) represents the singular value decomposition (SVD) to obtain the covariance matrix of ${\mathbf{X}}$; ${\mathbf{A}}$ = the orthogonal matrix composed of *m* eigenvectors. The diagonal elements of the diagonal matrix ${{\varvec{\Sigma}}}$ are the eigenvalues of each column of the orthogonal matrix, that is, the weight of each principal component. The eigenvectors of the orthogonal matrix ${\mathbf{A}}$ are arranged from large to small according to their corresponding eigenvalues. The *n* × *m* matrix composed of the first *n* columns of eigenvectors is the transformation matrix ${\mathbf{\rm T}}$. In the principal component analysis, the principal components are arranged in order of variance. When analyzing problems, some principal components can be discarded and only a few principal components with larger variance can be used to represent the original variables, thus reducing the calculation workload. When the principal component analysis method is used for a comprehensive evaluation, the cumulative contribution rate of the selected principal components is supposed to be greater than 85%. So even if a few principal components are used, the credibility is very high and the problem can also be explained effectively. Moreover, the selected principal components are uncorrelated with each other which eliminates multicollinearity. Based on the above characteristics, the principal component has better evaluation performance than the original indexes. The proportion of each principal component eigenvalue in the total eigenvalue of the extracted *p* principal components is taken as the weight to make a comprehensive evaluation. The calculation formula is as follows:

7 $$F = \eta_{1} f_{1} + \eta_{2} f_{2} + \cdots + \eta_{p} f_{p}$$

where

8 $$\eta_{k} = \lambda_{k} /\sum\limits_{i = 1}^{p} {\lambda_{i} }$$

9 $$f_{k} = \alpha_{k1} Z_{1} + \alpha_{k2} Z_{2} + \cdots + \alpha_{km} Z_{m}$$

In the formula, $\lambda_{1}$, $\lambda_{2}$,…,$\lambda_{p}$ = the extracted feature roots arranged in descending order; $f_{k}$ = *k*th principal components, *k* = 1, 2, …, *p*; $\alpha_{k}$ = $\left( {a_{k1} ,a_{k2} , \ldots ,\alpha_{km} } \right)^{{\text{T}}}$ = the eigenvector corresponding to the *k*th eigenvalue; $Z_{i}$ = original characteristic index after standardization, *i* = 1, 2, …, *m*; The purpose of comprehensive evaluation can be achieved by calculating and comparing the scores of each sample according to the model. The parameters of six characteristic indexes of the impedance spectrum curve after principal component analysis and coefficients of the six principal components are shown in Table 5.

**Table 5:** Principal component analysis of six indexes and coefficients of the six principal components.

Component	Eigen value	Variance (%)	Cumulative explained variance (%)	1# Index (*Z*_1_)	2# Index (*Z*_2_)	3# Index (*Z*_3_)	4# Index (*Z*_4_)	5# Index (*Z*_5_)	6# Index (*Z*_6_)
1	150,294.89	97.1480	97.1480	0.0270	0.0001	− 0.0019	− 0.9844	0.0202	0.1729
2	3216.74	2.0792	99.2273	− 0.1873	− 0.0006	0.0205	− 0.1688	− 0.3886	− 0.8860
3	1179.76	0.7626	99.9899	− 0.0876	− 0.0008	− 0.1140	0.0499	− 0.9028	0.4023
4	9.39	0.0061	99.9959	0.8496	− 0.0027	− 0.4975	− 0.0042	− 0.0875	− 0.1519
5	5.53	0.0036	99.9995	− 0.4845	− 0.0103	− 0.8596	− 0.0061	0.1610	0.0131
6	0.77	0.0005	100.0000	0.0029	− 0.9999	0.0103	0.0001	− 0.0004	0.0004

**Figure 13:**
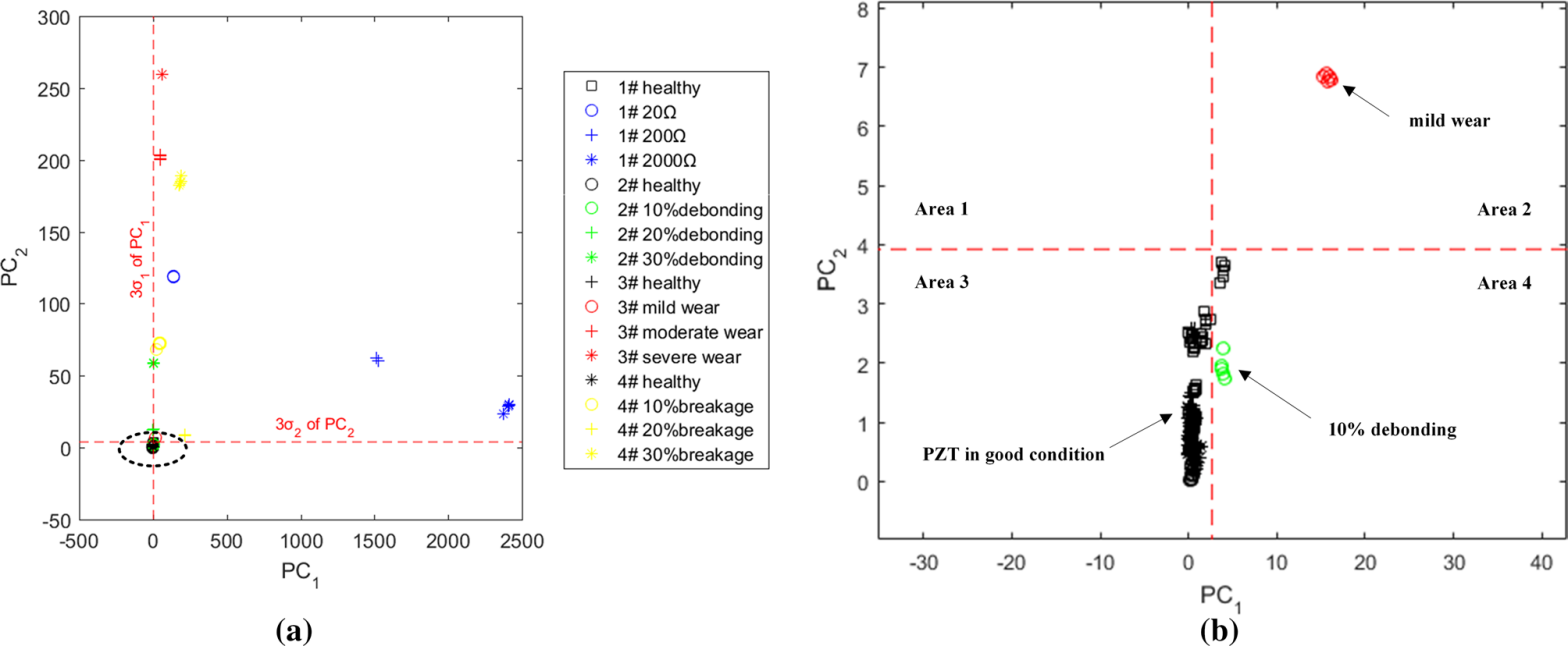
The effect of identification of damaged PZT with principal component analysis (**a**) and enlarged image of the elliptical area surrounded by a dashed line (**b**).

Table 5 shows that the cumulative variance contribution rate of the first two principal components is 99.2% and the first two principal components can better replace the above six indexes to judge whether the PZT faults occur. The first principal component is determined by the 4# index and the second one is determined by the 6# index. The first component reflects 97.1% of the original data information. After the principal component analysis, we take the first principal component as the abscissa and the second one as the ordinate. A total of 260 data with single structural damage or PZT fault are classified. The results of all data classification and identification are shown in Fig. 13a,b shows an elliptical area enlarged view. Due to the normal distribution of the normalized data, about 99.7% of the data points under the undamaged condition of PZT will be distributed within the range of 3 σ from the average value, that is, Area 3 in the lower-left corner surrounded by the red dotted line. When the data points appear in other areas, they can be identified as the damage of PZT. Figure 13a shows that there are obvious distinguishing effects between structural damages and PZT faults. With the aggravation of the sensor faults level, the offset between the data points and the average value is increasing, which shows that the method has a certain ability to reflect the degree of damage. The observation from Fig. 13b showed that the black data points representing the intact PZT are mainly concentrated in Area 3 while 5 of them are located in Area 4. In other words, there are only 5 data classification errors in 200 data and the classification accuracy of the method is 97.5%. Besides, compared with other damage conditions, the data points of 10% debonding are closest to Area 3 from which we can conclude that the slight debonding between PZT and the structure may likely be disregarded as structural damage in some situations.

**Figure 14:**
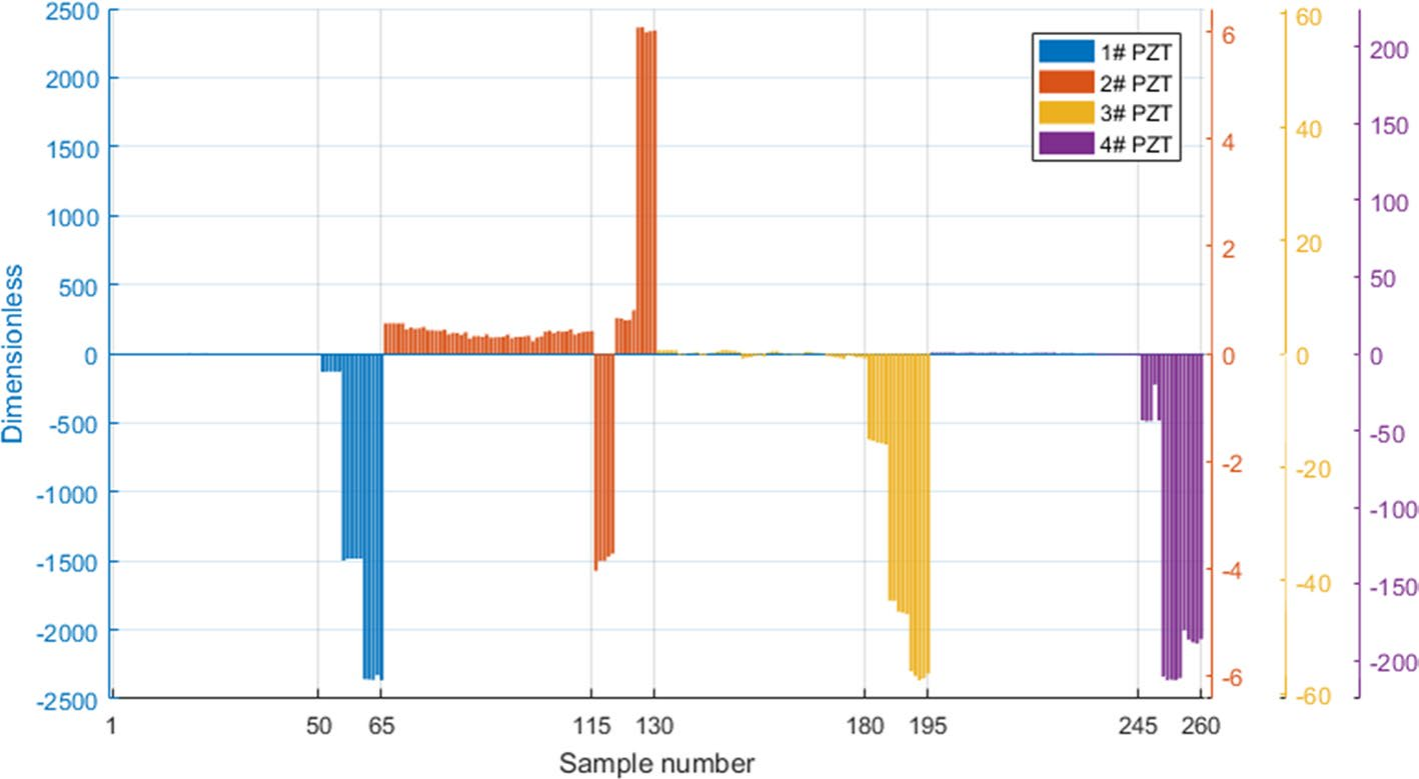
Histogram of the comprehensive principal component score.

The results of the principal component comprehensive evaluation of the data under various working conditions are shown in Fig. 14. Among all the samples 1–260, 1–65, 66–130, 131–195, and 196–260 were the four groups collected from 1–4# PZT. The first 50 data of each group is under the only structural damage conditions and the last 15 data is under the only PZT fault conditions. It is shown from Fig. 14 that there is a significant difference between the intact PZT and the damaged PZT. The absolute value of the former score is less than 0.5 while the latter is greater than 0.6. By comparing the comprehensive scores under various working conditions with the threshold value set in advance, PZT can be assessed accurately which further proves the feasibility of the principal component analysis method in judging the health status of PZT. To sum up, the PCA method can be used to judge the state of the structure and PZT no matter what cases and degrees of structural damages or sensor faults occur. It is of great significance for the actual identification of whether the PZT is damaged.

## Identification of different cases and degrees of PZT faults using LibSVM

After judging the damage condition of PZT with PCA, we often need to know more damage information, such as damage cases and damage degree. In this section, we use LibSVM to solve this multiclass classification problem.

LibSVM is first developed by Lin^46^ as a rapidly effective machine learning package for pattern recognition and regression. Its main idea is to establish a classification hyperplane as the decision surface, which maximizes the isolation edge between positive and negative examples and follows the principle of structural risk minimization. Four frequently-used kernel functions which are linear function, polynomial function, radial basis function (RBF), and S form function are provided for the multiclass problem, cross-validation to select parameters, the weighting of imbalance samples, and probability statistics of multiclass problem. As the advantage of open, extensibility, flexible use, and less parameter, the LibSVM is widely used in many producing and living activities. It can also be applied to the PZT self-fault identification and classification.

The specific steps of LibSVM are as follows:

1. Import external data and convert it to the format of the Support Vector Machine (SVM) package.
2. Simply amplify or narrow the data scale, the range of data after normalization is (0, 1].
3. Select the RBF kernel function $K(x,y) = e^{{ - \gamma \left\| {x - y} \right\|^{2} }}$.
4. Find the two best parameters c and g of the RBF kernel through cross-validation, c = the penalty coefficient which reflects the tolerance of error and its default value is 1, g = 1/k = the radius of kernel function which reflects the distribution of the data mapped to the new feature space (k is the number of attributes in the input data). The value of cross-validation is chosen as 2 by experience.
5. Train the SVM model with the best parameters and the training set.
6. Use the obtained model to classify the testing set.

When using the LibSVM for training, the raw data-set consists of a 260 by 6 matrix, in which the row number is 260 on behalf of the sample number and the column number is 6 on behalf of the number of attributes. The attributes refer to the above six principle components. Set “1” as the label for structural damage samples, “2”, “3”, “4” for pseudo soldering samples from mild to severe, “5”, “6”, “7” for 10% -30% debonding samples, “8”, “9”, “10” for wear samples from mild to severe, and “11”, “12”, “13” for 10%-30% breakage samples. Select 60% of each label data randomly as the training set and 40% as the testing set, so the training set is a 156 by 6 matrix and the testing set is a 104 by 6 matrix. For the improvement of classification precision, training samples should match with the label after mapping. And to examine the model furtherly, each parameter was tested by the testing samples.

The model trained with 156 sample data was used to classify and identify 104 samples under different working conditions and the results are shown in the Table 6. The identification is consistent with the labels which proves the feasibility of using LibSVM to accurately identify the cases and degree of PZT self-fault. It can be seen from the table that the recognition probability of label 1 is much higher than that of other labels, mainly because it includes larger training samples. With the increase of the training sample size, the recognition probability will be closer to 100%.

**Table 6:** PZT self-fault identification.

Label of testing data	The largest average probability	The second average probability	Other	Correct/total	Result
1	1 (86.7%)	2 (2.3%)	11.0%	80/80	1
2	2 (36.2%)	3 (11.1%)	52.7%	2/2	2
3	3 (35.2%)	4 (13.9%)	50.9%	2/2	3
4	4 (21.2%)	6 (15.4%)	63.4%	2/2	4
5	5 (35.3%)	4 (10.3%)	54.4%	2/2	5
6	6 (29.2%)	5 (10.9%)	59.9%	2/2	6
7	7 (33.1%)	8 (12.5%)	54.4%	2/2	7
8	8 (30.8%)	9 (10.4%)	58.8%	2/2	8
9	9 (29.5%)	10 (11.6%)	58.9%	2/2	9
10	10 (36.2%)	9 (11.1%)	52.7%	2/2	10
11	11 (30.8%)	12 (12.3%)	56.9%	2/2	11
12	12 (35.8%)	11 (14.5%)	49.7%	2/2	12
13	13 (30.6%)	12 (15.1%)	54.3%	2/2	13

## Summary and conclusions

For the long-term use of piezoelectric smart structures, it is difficult to distinguish the structural damage from the PZT self-fault with the intuitive changes of the impedance spectrum. To solve the problem, we proposed a new method of piezoelectric sensor self-diagnosis using principal component analysis and LibSVM. The method can judge whether the PZT is damaged and identify the cases and degrees of the damaged PZT which realizes the sensor self-diagnosis and greatly improve the ability of the electromechanical impedance method for SHM. The specific conclusions are as follows:

1. The sensitivity of the six characteristic indexes to four PZT faults is different and there are certain correlations between each index. The RMSD of reactance in the non-resonant frequency band and the area change rate of conductance in the resonance frequency band can extract the variation characteristics of the pseudo soldering’s levels, but it is insensitive to debonding and wear. At the same time, the slope of the susceptance linear fitting curve and the average shift of conductance peak frequency can only reflect the changes of wear and damage degree. Therefore, if only one or several indexes are used for evaluation, the judgment results will be one-sided and unstable. Additionally, the effective information extracted will be overlapped if the indexes are highly correlated.
2. Principal component analysis is used to judge whether the damage occurred on the structure or the PZT. The results show that the principal component analysis accurately identifies the damaged PZT after extracting two principal components and the accuracy reaches 97.5%. The 260 data absolute scores of PCA comprehensive evaluation were calculated and the scores of the intact sensors and sensors with fault are less than 0.5 and greater than 0.6, respectively. By calculating the score and comparing it with the set threshold, whether the PZT faults occur is judged.
3. When using LibSVM to identify the damage cases and degrees of PZT, the SVM model trained with 156 samples can all correctly classify the PZT state of 104 data into intact and 12 possible damage states which proves the feasibility of the method for sensor fault identification. As the size of the training set increases, the identification result is expected to be closer to 100% which indicates a better classification ability.

This method with strong operability and practical application value can be used in a large number of PZT transducers self-test scenarios and provide a reference for self-assessment of the health of other transducers e.g. ultrasonic transducers in SONAR/ medical. However, there are still many research directions worthy of further analysis, such as finding suitable characteristic indexes for all types of PZT faults, evaluating the detection ability of damaged PZT, and testing the applicability of the trained LibSVM model for signals measured under ambient environment changes.
